# Echinocystic Acid Inhibits Inflammation and Exerts Neuroprotective Effects in MPTP-Induced Parkinson’s Disease Model Mice

**DOI:** 10.3389/fphar.2021.787771

**Published:** 2022-01-19

**Authors:** Dewei He, Guiqiu Hu, Ang Zhou, Yanting Liu, Bingxu Huang, Yingchun Su, Hefei Wang, Bojian Ye, Yuan He, Xiyu Gao, Shoupeng Fu, Dianfeng Liu

**Affiliations:** ^1^ College of Animal Science, Jilin University, Changchun, China; ^2^ College of Veterinary Medicine, Jilin University, Changchun, China; ^3^ Department of Neurosurgery, Seoul St. Mary’s Hospital, College of Medicine, The Catholic University of Korea, Seoul, South Korea

**Keywords:** Parkinson’s disease, neuroinflammation, echinostic acid, microglia, NF-κB/MAPK

## Abstract

Parkinson’s disease (PD), the second primary neurodegenerative disease affecting human health, is mainly characterized by dopaminergic neuron damage in the midbrain and the clinical manifestation of movement disorders. Studies have shown that neuroinflammation plays an important role in the progression of PD. Excessively activated microglia produce several pro-inflammatory mediators, leading to damage to the surrounding neurons and finally inducing neurodegeneration. Echinocystic acid (EA) exhibits an anti-inflammatory effect in peripheral tissues. However, whether it inhibited neuroinflammation remains unclear. Therefore, the current study investigates the effect of EA on neuroinflammation and whether it can improve PD symptoms through inhibiting neuroinflammation. In our experiments, we discovered that EA inhibited the production of pro-inflammatory mediators in LPS-exposed BV2 cells. Further mechanism-related studies revealed that EA inhibited inflammation by activating PI3K/Akt and inhibiting NF-κB and MAPK signal pathways in LPS-induced BV2 cells. Research revealed that EA eases microglia-mediated neuron death in SN4741 and SHSY5Y cells. In *in vivo* studies, the results demonstrated that EA improves weight loss and behavioral impairment in MPTP-induced mice. Further studies have revealed that EA inhibited dopaminergic neuron damage and inflammation in the mice midbrain. In conclusion, our study demonstrated that EA inhibits neuroinflammation and exerts neuroprotective effects by activating PI3K/Akt and inhibiting NF-κB and MAPK signal pathways *in vivo* and *in vitro*.

## 1 Introduction

Parkinson’s disease (PD) is the second most prevalent neurodegenerative disorder, and its clinical manifestations are mainly motor symptoms such as resting tremor and muscle rigidity. Studies have shown that the combined effects of aging, genetics, and environmental factors lead to the onset of PD (Pollak, 2004; Nakano-Kobayashi et al., 2020; Ramli et al., 2020). However, the specific etiology of PD is not fully understood. At present, the commonly used clinical treatment methods mainly focus on rehabilitation training to assist drug therapy, but the rehabilitation training has little effect on severely ill patients. The commonly used clinical drugs have limited effects, and their long-term use has strong side effects (Bomalaski et al., 2017; Houman, 2018; Elkouzi et al., 2019). Therefore, exploring the pathogenesis of PD and discovering effective and alleviating drugs for PD treatment are required.

The main pathological features of PD are the formation of Lewy bodies and loss of dopaminergic neurons in the midbrain. An increasing number of evidence showed that the course of PD is accompanied by neuroinflammation, and excessive inflammation will in turn exacerbate PD. During the neuroinflammation process, immune cells are activated to release inflammatory mediators, damaging peripheral neurons. Long-term continuous damage leads to degeneration and loss of several dopaminergic neurons, exhibiting PD symptoms (Lee et al., 2009; Bartels and Leenders, 2010; Chung et al., 2010; Pereira et al., 2018). Microglia, the resident immune cells of the brain, are the main participants of neuroinflammation. The autopsy report also showed that several microglial cells were activated in the midbrain of patients with PD (Alstadhaug et al., 2017; Hopperton et al., 2017). Excessively activated microglia produce several pro-inflammatory mediators, leading to damage to surrounding neurons and finally inducing neurodegeneration. Therefore, inhibiting the excessive activation of microglia and the neuroinflammatory response involved might have a certain effect on alleviating PD.

Lipopolysaccharide (LPS) is a component of the cell wall of Gram-negative bacteria. Studies have shown that LPS stimulation can activate the inflammatory response in immune cells through Toll-like receptor 4 (TLR4) (Huang et al., 1995; Lu et al., 2008). In this experiment, we treated BV2 cells with LPS to induce an *in vitro* microglia inflammation model. 1-Methyl-4-phenyl-1, 2, 3, 6-tetrahydropyridine hydrochloride (MPTP) is a lipophilic toxin that can penetrate the blood–brain barrier. After entering the brain, MPTP is converted to 1-methyl-4-phenylpyridine (MPP^+^) by monoamine oxidase B and damages neurons (Kan et al., 1984; Przedborski et al., 1996). In the experiment, we injected MPTP into the abdominal cavity to construct a PD mouse model.

Echinocystic acid (EA), derived from *Albizzia julibrissin* Durazz (Fabaceae) inflorescence (Shao et al., 1996; Dhaneshwar et al., 2010; Liu et al., 2017), exhibits a spectrum of pharmacological effects, such as liver and blood nourishment, blood coagulation, and immune regulation activity. As a natural compound, EA has the advantages of small side effects and easy availability. Therefore, EA effects are widely studied in various diseases. Recently, many studies have shown that EA exhibits excellent anti-inflammatory, antioxidant, and antitumor effects (Jæger et al., 2017; Fu et al., 2018). Studies found that EA inhibits macrophage inflammation and improves LPS-induced acute lung injury in mice *via* the NF-κB and MAPK pathways (Joh et al., 2012; Deretic, 2021). Studies have also revealed that EA alleviates cerebral ischemia/reperfusion injury by inhibiting the JNK signaling pathway (Yu et al., 2019). In addition, EA can inhibit cerebral ischemia *via* the PI3K/Akt pathway in intracerebral hemorrhage mice (Chen et al., 2020). We assume that EA might inhibit neuroinflammation and exert a neuroprotective effect in PD based on the aforementioned findings.

## 2 Methods and Materials

### 2.1 Reagents

Echinocystic acid (EA, >98.0% purity; Shanghai Yuan-ye, St. Louis, Shanghai, China), lipopolysaccharide (LPS, Sigma, St. Louis, MO, United States), dimethyl sulfoxide (DMSO), LY294002 (a PI3K inhibitor), and MK2206 (an Akt inhibitor) were obtained from Sigma Aldrich (St Louis, MO, United States). Dulbecco’s modified Eagle’s medium (DMEM) and fetal bovine serum (FBS) were obtained from Gibco (Grand Island, NY, United States). Penicillin–streptomycin (PS) solutions were obtained from Invitrogen (Carlsbad, CA, United States). Trypsin was obtained from Beyotime Institute (Biotech, Beijing, China).

### 2.2 Cell Culture and Treatment

BV2 (murine microglia cell line), SN4741 (murine dopaminergic neuron cell line), and SHSY5Y (human neuroblastoma cell line) were obtained from Shanghai Binsui Biological Technology (Shanghai, China). The cultured conditions of cells were as follows: 89% DMEM+10% FBS+1% penicillin–streptomycin (PS) solutions, 95% air, 5% carbon dioxide (CO2), and a temperature of 37°C. The cells were digested with 0.25 and 0.05% trypsin, and passaged every 2 days. The cells were seeded into a 6-cm diameter cell culture dish during the research period. When the density was 70–80%, the medium was replaced with serum-free DMEM, and then EA was added to the petri dish and incubated for 1 h. Later, the cells were exposed to LPS (100 ng/ml) for 1, 12, and 24 h. During the experiment, SN4741and SHSY5Y cells were cultured in a conditioned medium.

### 2.3 CCK-8 Assay

Cells were seeded into 96-well plates at a density of 1–2 × 10^4^ cells per well and cultured overnight in a cell incubator. When the density was moderate, different concentrations of EA and solvent DMSO were added to the culture wells and then cultured. After 24 h, the culture medium was replaced with CCK8 dilution (Beyotime Inst Biotech, Beijing, China) and incubated for 2 h. After that, cell viability was measured with a microplate reader at 450 nm.

### 2.4 LDH Assay

Cells were seeded into 96-well plates at a density of 1–2 × 10^4^ cells per well and cultured overnight in a cell incubator. Different concentrations of EA and solvent DMSO were added to the culture wells, and then the cells were cultured. After 24 h, the release of LDH in the medium was determined using an LDH assay kit (Beyotime Inst Biotech, Beijing, China) according to the manufacturer’s instructions.

### 2.5 Quantitative PCR

According to the manufacturer’s protocols, cells and tissue RNAs were extracted using the Trizol reagent (Invitrogen, Carlsbad, CA, United States). After determining the concentration, 2 μg RNA was reverse-transcribed into cDNA using a reverse transcription kit (Sigma-Aldrich, St. Louis, MO, United States). Then quantitative PCR (qPCR) was performed using the SYBR Green QuantiTect RT-PCR Kit (Invitrogen, Carlsbad, CA, United States), according to the manufacturer’s protocols. The mRNA levels of inflammatory mediators were evaluated relative to β-actin according to the 2^−ΔΔCT^ method. The primers involved in the experiment are presented in Table 1.

**TABLE 1:** Primer sequences for quantitative PCR.

Gene	Sequences
*IL-6*	(F) 5′-CCA​GAA​ACC​GCT​ATG​AAG​TTC​C-3′
	(R) 5′-GTT​GGG​AGT​GGT​ATC​CTC​TGT​GA-3′
*TNF-α*	(F): 5′-ACG​GCA​TGG​ATC​TCA​AAG​AC-3′
	(R): 5′-GTG​GGT​GAG​GAG​CAC​GTA​GT-3′
*iNOS*	(F) 5′-GAA​CTG​TAG​CAC​AGC​ACA​GGA​AAT-3′
	(R) 5′-CGT​ACC​GGA​TGA​GCT​GTG​AAT-3′
*COX-2*	(F) 5′-CAG​TTT​ATG​TTG​TCT​GTC​CAG​AGT​TTC-3′
	(R) 5′-CCA​GCA​CTT​CAC​CCA​TCA​GTT-3′
*β-actin*	(F): 5-GTC​AGG​TCA​TCA​CTA​TCG​GCA​AT-3
	(R): 5-AGA​GGT​CTT​TAC​GGA​TGT​CAA​CGT-3

### 2.6 ELISA

When the cells seeded into a 24-well plate were 70–80% in density, the medium was changed to serum-free DMEM. Then the cells were incubated with EA (1 h, 16 μM) and stimulated with LPS (100 ng/ml, 24 h). After that, the expression of IL-6 and TNF-α in the supernatant was measured using the ELISA kits, according to the manufacturer’s protocols (BioLegend, San Diego, CA, United States).

### 2.7 Western Blot

Total protein of BV2 cells and mice midbrain tissue was extracted using P0013 Lysis Solution (Beyotime Ist. Beijing, China). Protein concentration was measured using a BCA reagent (Beibo Biological Technology, Shanghai, China). The 30 µg protein was loaded and separated by 13% SDS-PAGE, and then transferred to polyvinylidene difluoride (PVDF) membranes (Biyuntian Biological Reagent, Beijing, China). After blocking with 5% skim milk for 2 h at room temperature, the PVDF membranes were incubated with primary antibodies [COX-2 (1:5,000), iNOS (1:5,000), p-Akt (1:5,000) (Abcam, Cambridge, United Kingdom), p-JNK1/2 (1:5,000), p-ERK1/2 (1:5,000) (Cell Signal Technology, MA, United States), p-p38 (1:5,000), p-NF-κB p65 (1:5,000), JNK1/2 (1:5,000), ERK1/2 (1:5,000), p38 (1:5,000), Akt (1:5,000), NF-κB p65 (1:5,000), and β-actin (1:5,000) (Santa, CA, United States)] for 12 h at 4°C and then washed three times (20 min per time) with TBST solution. Thereafter, the membranes were incubated with the following secondary antibodies: goat anti-rabbit (1:5,000) and goat anti-mouse (1:5,000) (Santa, CA, United States) for 2 h, and then washed three times (20 min per time) with TBST solution. All the primary antibodies and secondary antibodies were dissolved in a 5% BSA solution. Next, the membranes were incubated with ECL Luminous Liquid (Amersham Pharmacia Biotech, Philadelphia, United States), and the target proteins were presented with the ScanLater Western Blot Detection System (Meigu Molecular Instruments, Shanghai, China).

### 2.8 Animals and Treatment

Forty male C57BL6 mice (25–30 g) were purchased from the Experimental Animals Center of Norman Bethune Medical College of Jilin University (Changchun, PR China). Animals were kept at 22–24°C in an artificial 12/12 h day/night cycle condition. All the animals were provided with adequate food and drink. Animal models were induced with 1-methyl-4-phenyl-1, 2, 3, 6-tetrahydropyridine hydrochloride (MPTP, Sigma-Aldrich, MO, United States). The mice were randomly divided into three groups during the experiment: saline, MPTP, and MPTP + EA. MPTP (30 mg/kg, dissolved in saline) was injected intraperitoneally for 7 d. EA (dissolved in saline) was administered intragastrically once a day for 17 days. After administration, the mice were tested through behavioral tests. After that, the mice were euthanized.

### 2.9 Behavioral Tests

#### 2.9.1 Open Field Test

The mobility of mice was tested through the open field test. In the test, mice were placed into the box (50 × 50 × 30 cm, Any-maze, Stoelting Co.) for 5 min. During this time, the total distance mice traveled and the time mice spent in the inner zones of the box were recorded with an overhead camera and analyzed using Top Scan software (Any-maze, Stoelting Co.), according to the manufacturer’s instructions. We maintained a quiet environment during the experiment.

#### 2.9.2 Climbing Pole Experiment

The mice were placed on the top of a wooden pole with a height of 60 cm and a diameter of 1 cm for the experiment of climbing the pole. The timing began when the hind legs of the mice left the top of the pole, and the timing stopped when the mice landed on the ground. The experiment was repeated three times for each mouse and the average value was recorded. The mice were trained to climb the pole every day for 1 week before the experiment.

#### 2.9.3 Turn bar Experiment

The mice were put on the rotor-rod fatigue instrument, the rotor-rod speed was adjusted so that the normal mice stayed on the rotor-rod for 60–90 s, and the rotor-rod speed was recorded as the setting value during the experiment. During the experiment, mice were placed on the rotary-rod fatigue instrument, the instrument was started, and the residence time of mice on the rotary-rod was recorded by timing. Each mouse was put on the instrument three times, and the average value (residence time) was recorded. The mice were trained daily for 1 week before the experiment.

### 2.10 Immunohistochemical Staining

The number of dopaminergic neurons and microglia activation in the substantia nigra of the mouse midbrain were detected through immunohistochemical staining. In brief, after being euthanized, the midbrains of mice were soaked in a 4% paraformaldehyde solution for 24 h, and then processed using the following procedure: alcohol (70, 80, 90% each for 1 h, 100% for 2 h), xylene (20 min), and then dipping wax (40 min). Then the tissues were sectioned at 5 µm per slice. Thereafter, the immunohistochemistry staining was performed with an UltrasensitiveTM S-P kit (MBX Biotechnologies, Fuzhou, China), according to the manufacturer’s protocols. The dopaminergic neurons were marked with the anti-TH and anti–IBA-1 antibodies (1:200, Abcam, Cambridge, United Kingdom). IHC images were taken under an optical microscope. Total TH-positive cells and IBA-1–positive cells were counted by five researchers blind to the experimental design, and the average of these scores was reported.

### 2.11 Statistical Analysis

Differences in data were analyzed using SPSS 19.0 software (IBM). All data were represented as means ± SD. Differences of groups were calculated adopting a one-way ANOVA plus multiple testing method and controlling the within-group error. Differences were considered statistically significant at *p <* 0.05 and *p <* 0.01.

## 3 Results

### 3.1 The Effect of EA on the Viability of BV2 Cells

**FIGURE 1:**
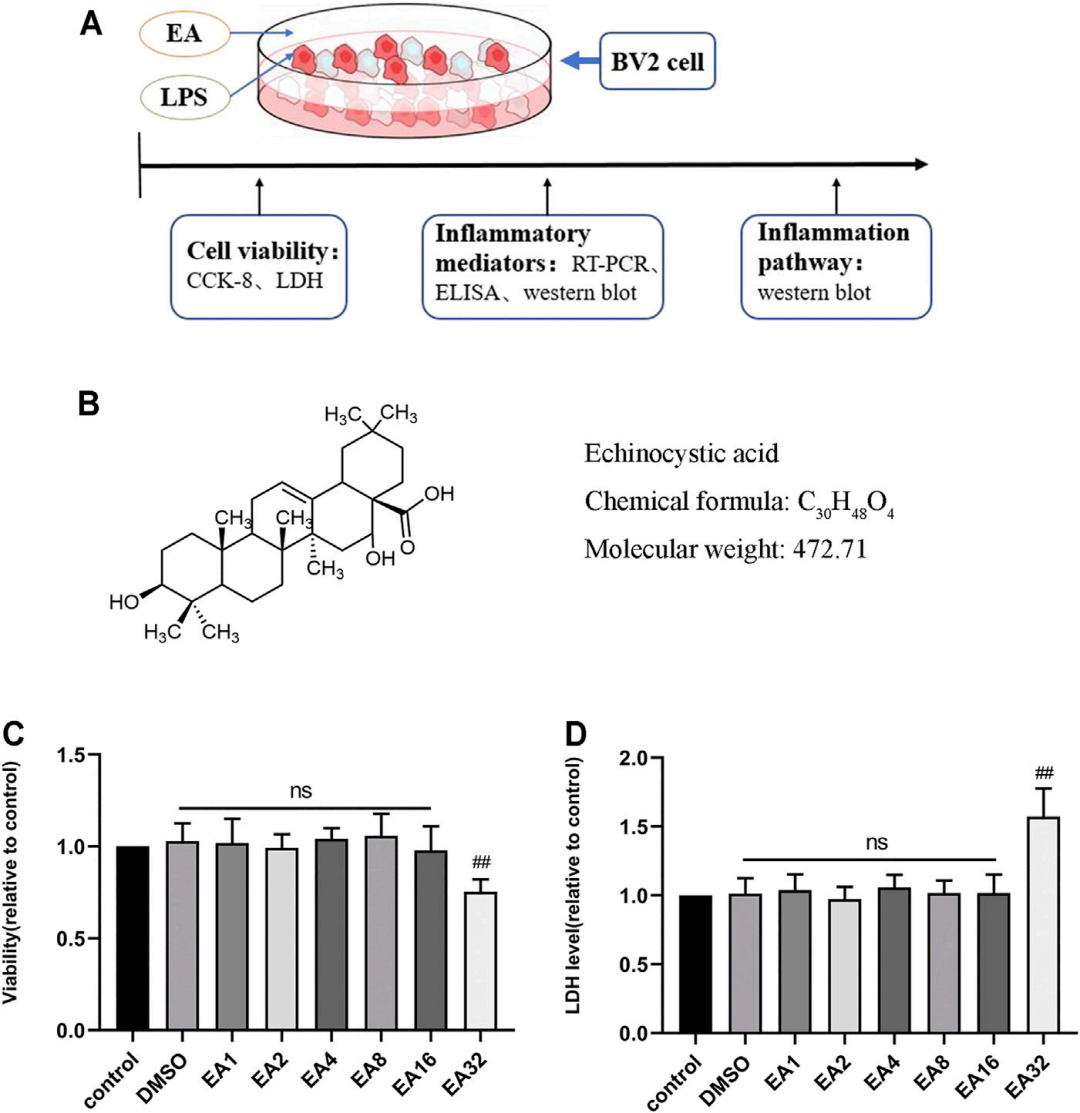
Effect of EA on the viability of BV2 cells. **(A)** Processing of BV2 cells. **(B)** The structure of EA. **(C)** The survival rate of BV2 cells treated with different concentrations of EA (1, 2, 4, 8, 16, and 32 μM) for 24 h *via* CCK-8 experiment. **(D)** Effects of EA on LDH release in BV2 cells. Results are represented as means ± SD (*n* = 4). ^*##*^ *p <* 0.01 vs. control group.

To determine whether EA affects the growth of BV2 cells, we tested the effect of different concentrations of EA (0–32 μM) on the survival rate of BV2 cells and LDH release. The results of CCK8 showed that EA no more than 16 μM did not affect the survival rate of BV2 cells (Figure 1C). The results of LDH showed that EA no more than 16 μM did not affect the LDH release of BV2 cells (Figure 1D). These results demonstrated that EA no more than 16 μM did not show potential toxic effects on BV2 cells.

### 3.2 EA Inhibits the Production of Inflammatory Mediators

**FIGURE 2:**
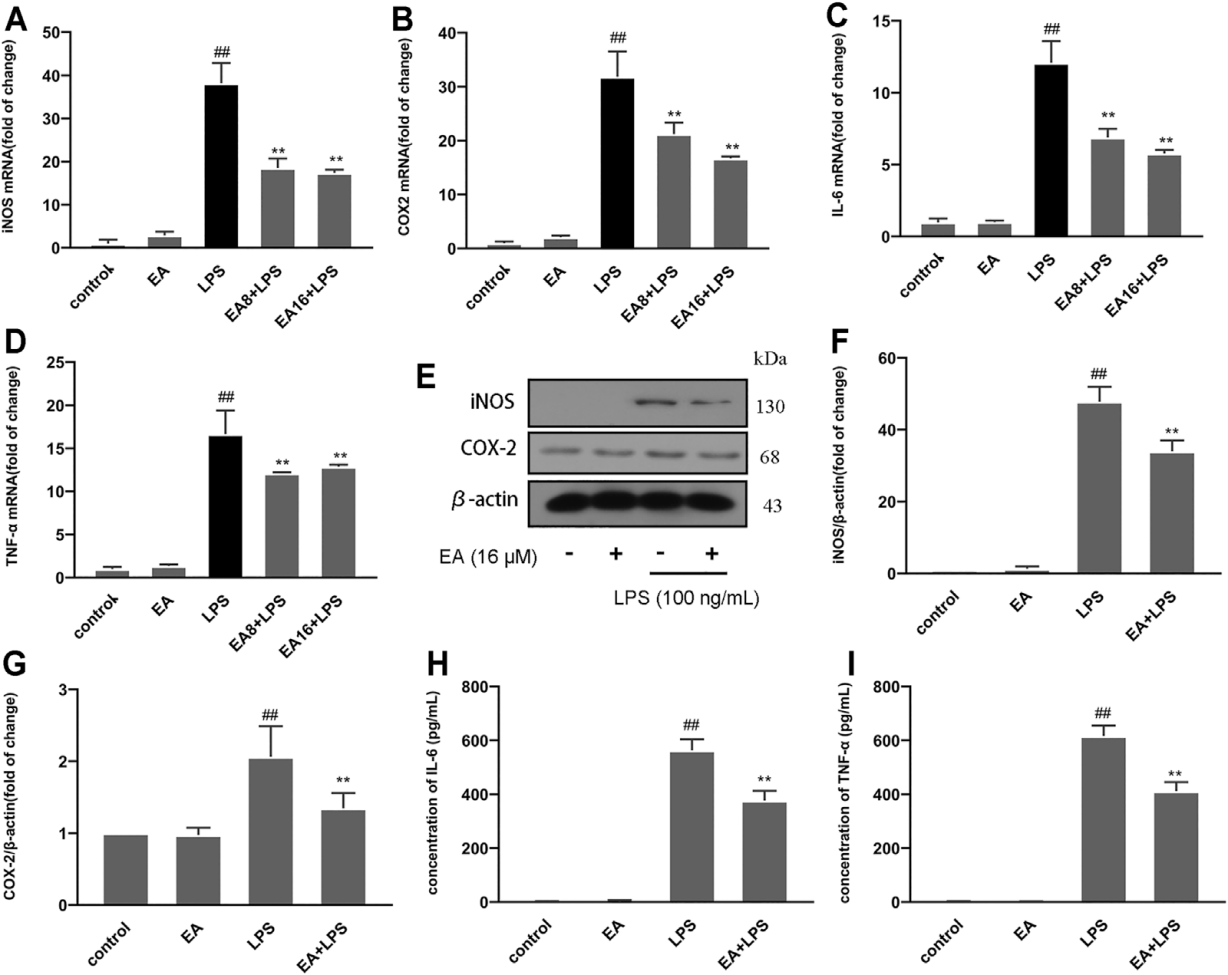
EA inhibits the production of inflammatory mediators. We treated BV2 cells for 1 h with EA (8 and 16 μM) and stimulated for 12 h with LPS (100 ng/ml). Then we examined the effect of EA on mRNA expression of inflammatory mediators [iNOS **(A)**, COX-2 **(B)**, IL-6 **(C)**, and TNF-α **(D)**] *via* quantitative PCR experiment. We treated BV2 cells for 1 h with EA (16 μM) and stimulated for 24 h with LPS (100 ng/ml). Then we examined the effect of EA on protein expression of inflammatory mediators [iNOS **(E,F)**, COX-2 **(E,G)**, IL-6 **(H)**, and TNF-α **(I)**] *via* Western blot and ELISA experiment. Results are represented as means ± SD (*n* = 4). ^*##*^ *p < 0.01* vs. control group. **p < 0.01 vs. LPS-exposed group.

When inflammation occurs, immune cells release a large amount of pro-inflammatory factors (IL-6 and TNF-α) and pro-inflammatory enzymes (iNOS and COX-2), which are considered one of the causes of damage to the surrounding tissues and neurons. To clarify the anti-inflammatory effect of EA, we treated cells with EA (16 μM) for 1 h and stimulated them with LPS (100 ng/ml) for another 12 (mRNA) or 24 h (protein). The mRNA expression of pro-inflammatory mediators [iNOS (Figure 2A), COX-2 (Figure 2B), IL-6 (Figure 2C), and TNF-α (Figure 2D)] was measured by the quantitative PCR method, and the protein expression was measured by ELISA (IL-6 (Figure 2H), TNF-α (Figure 2I), and Western blot [iNOS (Figures 2E,F) and COX-2 (Figures 2E,G)] technology. Results showed that EA inhibited the mRNA and protein expression of inflammatory mediators.

### 3.3 EA Inhibits Inflammation Through the PI3K/Akt Signal Pathway in BV2 Cells

**FIGURE 3:**
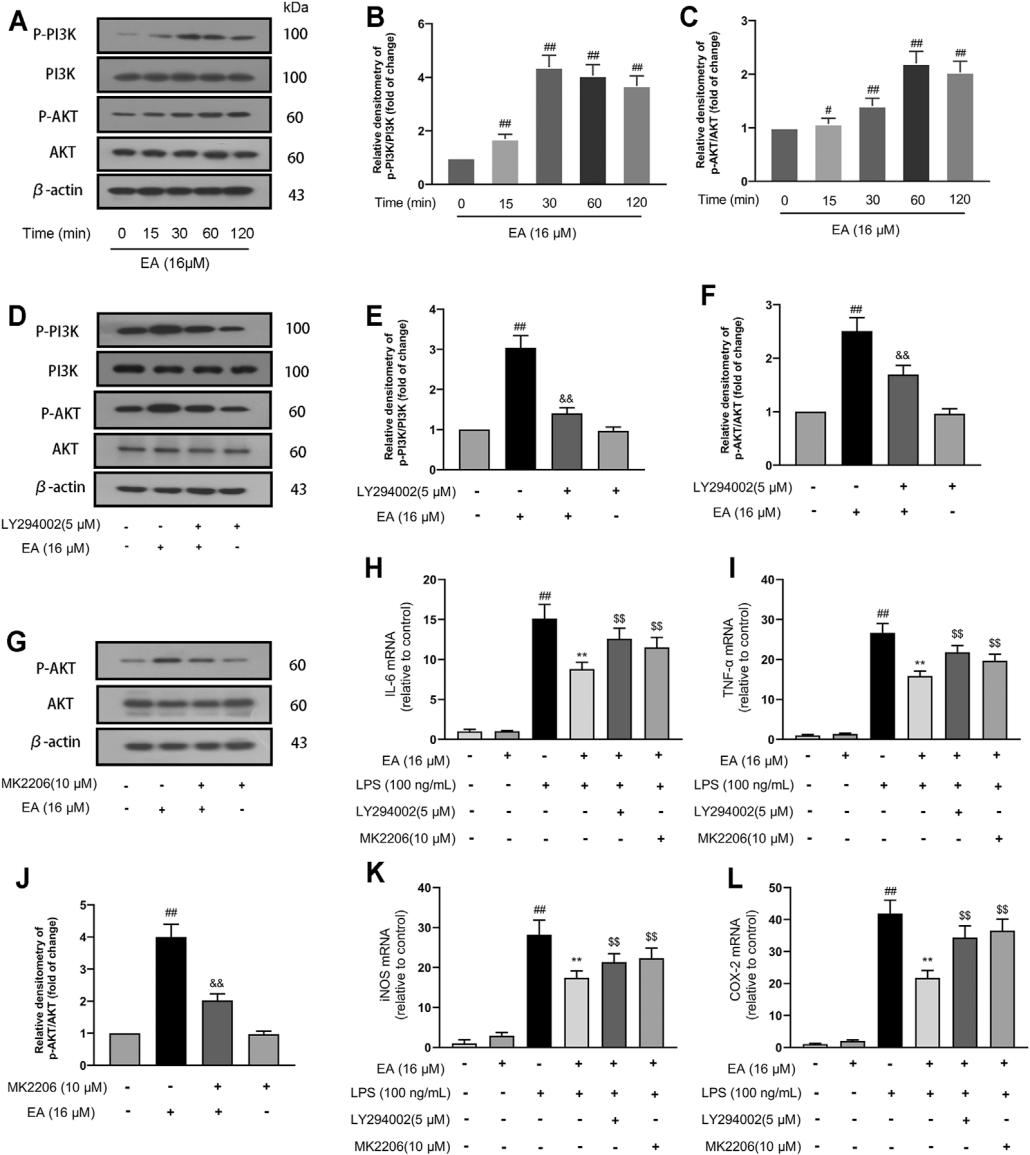
EA inhibits inflammation through the PI3K/Akt signal pathway. We treated BV2 cells for 1 h with EA (16 μM) and stimulated for 12 h with LPS (100 ng/ml). BV2 cells were treated for different times (0, 15, 30, and 60 min) with EA (16 μM), and then the protein expression of p-PI3K, PI3K, p-Akt, Akt, and β-actin was examined *via* Western blot experiment **(A–C)**. BV2 cells were treated for 6 h with LY294002 (a PI3K pathway inhibitor, 5 μM) or MK2206 (an Akt pathway inhibitor 10 μM), and then the protein expression of p-PI3K, PI3K, p-Akt, Akt, and β-actin was examined *via* Western blot experiment **(D–H)**. Thereafter, we treated BV2 cells for 1 h with EA (16 μM) and stimulated for 12 h with LPS (100 ng/ml), and then surveyed the effect of EA on the mRNA levels of inflammatory mediators [iNOS **(K)**, COX-2 **(L)**, IL-6 **(I)**, and TNF-α **(J)**] *via* quantitative PCR experiment. Results are represented as means ± SD (*n* = 4). ^*##*^ *p <* 0.01 vs. control group. **p < 0.01 vs. LPS-exposed group. ^$$^ *p < 0.01* vs. LPS + EA group.

The PI3K/Akt is an intracellular signal transduction pathway responsible for cell proliferation and survival. In the experiment, we investigated the effect of EA on the PI3K/Akt signal pathway. We treated BV2 cells at different times (0, 15, 30, and 60 min) with EA (16 μM) and measured the phosphorylation changes of PI3K/Akt signal pathways. Results showed that EA promoted the activation of PI3K (Figures 3A,B) and Akt (Figures 3A,C) pathway in the BV2 cells. Thereafter, we treated BV2 cells with PI3K pathway inhibitors (LY294002, 5 μM) for 6 h and measured the activation of the PI3K/Akt pathway. Results showed that LY294002 could block the activation of PI3K (Figures 3D,E) and Akt (Figures 3D,F) pathway by EA to a certain extent. Similarly, Akt pathway inhibitor (MK2206, 10 μM for 6 h) treatment inhibited the activation of the Akt pathway by EA (Figures 3G,H).

In further studies, we treated BV2 cells with pathway inhibitors (LY294002 or MK2206) for 6 h and measured the expression of pro-inflammatory mediators. The results showed that treatment with pathway inhibitors inhibited the regulatory effect of EA on inflammatory mediators [iNOS (Figure 3K), COX-2 (Figure 3L), IL-6 (Figure 3I), and TNF-α (Figure 3J)] in LPS-exposed BV2 cells. These results demonstrated that EA inhibits inflammation through the PI3K/Akt signal pathway.

### 3.4 EA Inhibits NF-κB and MAPK Pathways in BV2 Cells

**FIGURE 4:**
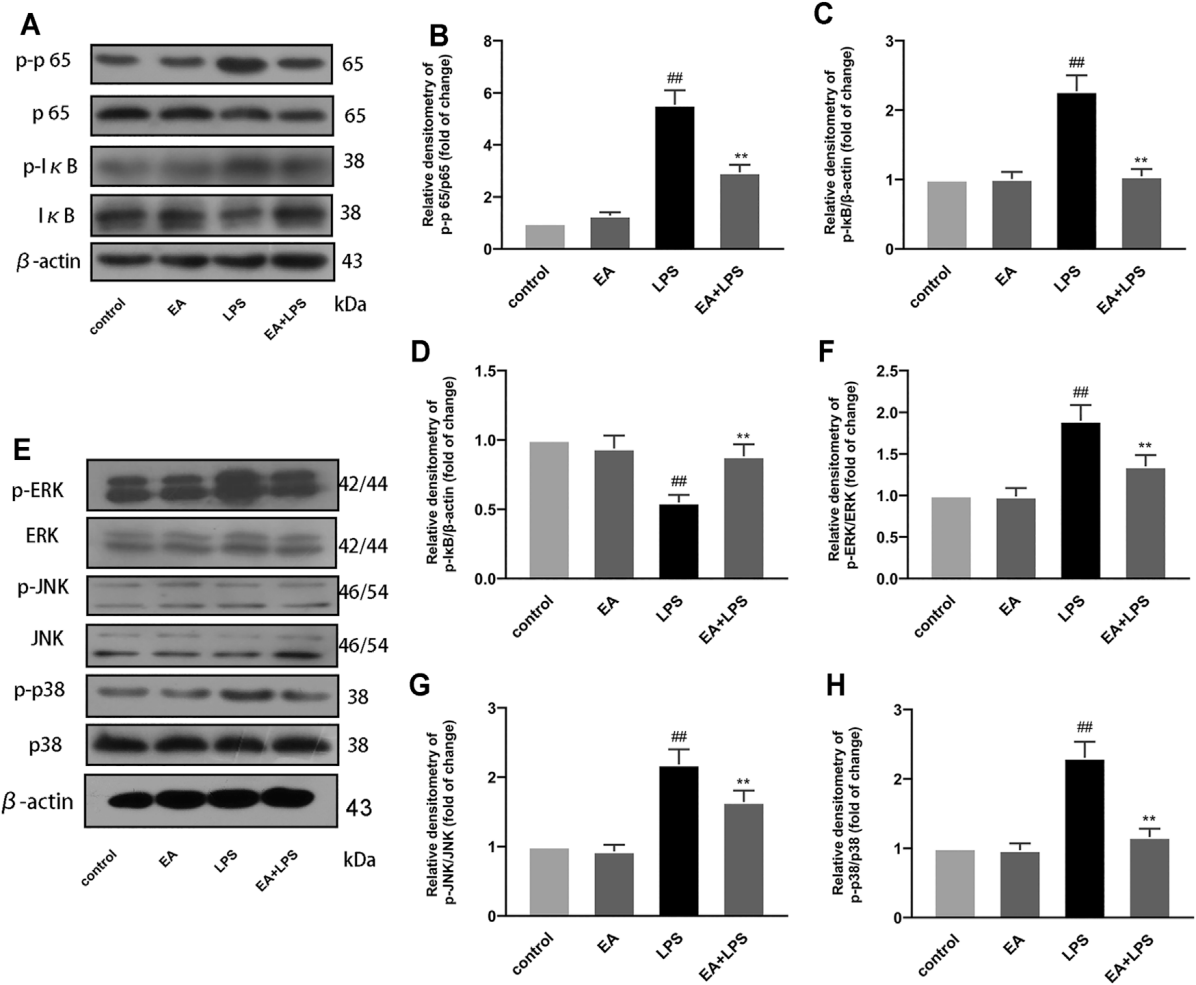
EA inhibits NF-κB and MAPK signal pathway. We treated BV2 cells for 1 h with EA (16 μM) and stimulated for 2 h with LPS (100 ng/ml). Then we examined the effect of EA on the protein expression of p-p65, p65 **(A,B)**, p-IκB **(A,C)**, IκB **(A,D)**, p-ERK, ERK **(E,F)**, p-JNK, JNK **(E,G)**, p-p38, p38 **(E,H)**, and β-actin *via* Western blot experiment. Results are represented as means ± SD (*n* = 4). ^*##*^ *p <* 0.01 vs. control group. **p < 0.01 vs. LPS-exposed group.

Studies have revealed that NF-κB and MAPK signal pathways play a crucial role in the process of inflammation, and their activation can regulate the transcription of inflammatory mediators. In order to clarify the mechanism of EA regulating inflammatory mediators, we studied the effect of EA on NF-κB and MAPK pathway activation. We treated the cells with EA (16 μM) for 1 h and then stimulated with LPS (100 ng/ml) for another 1 h. Then we detected the phosphorylation levels of NF-κB p65, IκBα, ERK, JNK, and p38 and their expression by Western blot. Results proved that EA inhibited the phosphorylation of NF-κB p65 (Figures 4A,B), IκBα (Figures 2 A,C), ERK (Figures 4 E,F), JNK (Figures 4E,G), and p38 (Figures 4 E,H), and the degradation of IκBα (Figures 4 A,D). These results demonstrated that EA inhibited the activation of NF-κB and MAPK signal pathways in LPS-exposed BV2 cells.

### 3.5 EA Eases Microglia-Mediated Neuron Death in SN4741 and SHSY5Y Cells

**FIGURE 5:**
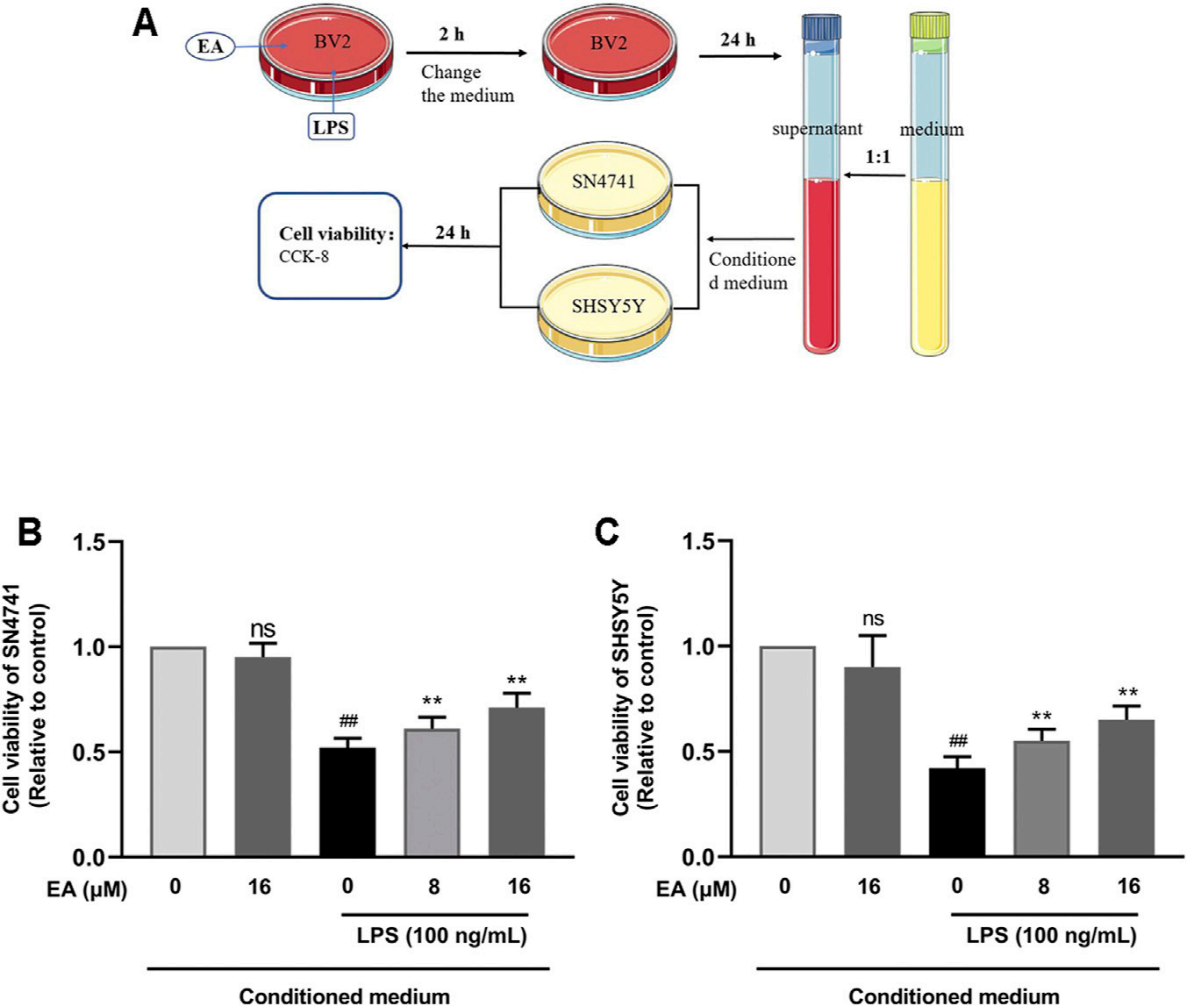
EA eases microglia-mediated neuron death in SN4741 cells and SHSY5Y cells. We treated BV2 cells with EA (16 μM) and LPS (100 ng/ml) for 2 h, and then changed the medium. After 24 h, the medium was collected and mixed with the fresh medium in a 1:1 ratio as the conditioned medium **(A)**. Then the effect of the conditioned medium on the survival rate of SN4741 cells **(B)** and SHSY5Y cells **(C)** was detected by CCK8 experiment. Results are represented as means ± SD (*n* = 4). ^*##*^ *p < 0.01* vs. control group. **p < 0.01 vs. LPS-exposed group.

To study the neuroprotective effect of EA on neural cells, we cultured two types of neuron cell lines (SN4741 and SHSY5Y). We incubated BV2 cells with EA (16 μM) and LPS (100 ng/ml) for 2 h and then changed the medium. After 24 h, the medium was collected and mixed with fresh medium in a 1:1 ratio as the conditioned medium (Figure 5A). When the density of SN4741 and SHSY5Y cells was 50–60%, the medium was changed to a conditional medium, and then the survival rate of cells was detected through CCK8 experiment. Results showed that EA eases microglia-mediated neuron death in SN4741 (Figure 5B) and SHSY5Y cells (Figure 5C).

### 3.6 EA Improves MPTP-Induced Body Weight Loss and Locomotor Activity Decreasing in a Mouse Model of PD

**FIGURE 6:**
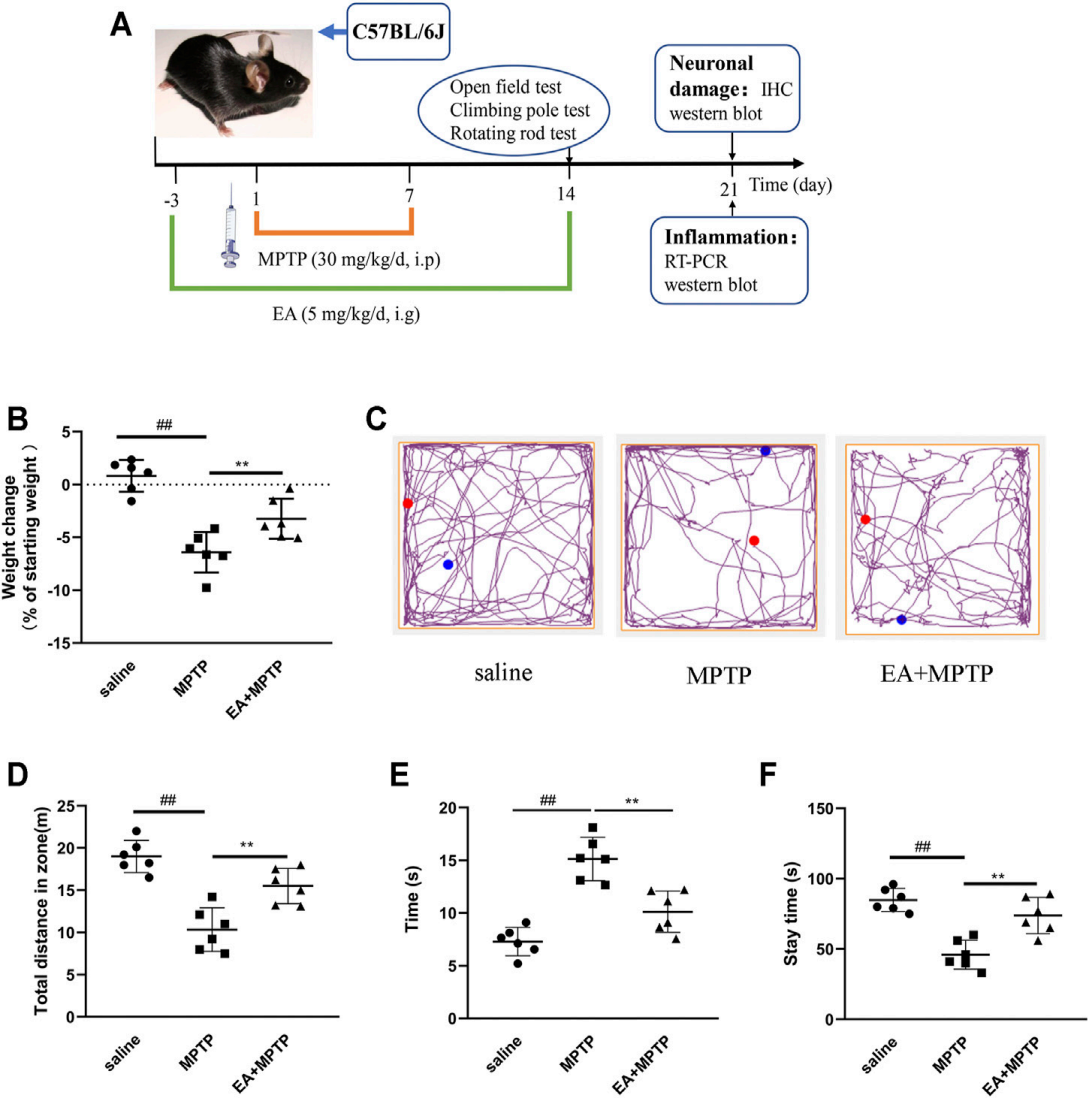
EA improves MPTP-induced body weight loss and locomotor activity decreasing in a mouse model of PD. Mice were given EA (5 mg/kg) by gavage for 17 d and MPTP (30 mg/kg) by intraperitoneal injection for 7 d **(A)**. Then we examined the effect of EA on body weight loss **(B)** of mice and the locomotor activity *via* open-field **(C,D)**, rod climbing **(E)**, and rod rotating **(F)** experiments. Results are represented as means ± SD (*n* = 6). ^*##*^ *p <* 0.01 vs. saline group. **p < 0.01 vs. MPTP group.

The main clinical manifestation of PD is decreased or loss of locomotor activity. We detected the effects of EA on weight loss and locomotor activity in MPTP-induced PD mice. Results showed that EA (5 mg/kg) improved MPTP-induced body weight loss (Figure 6B). Open-field experiments showed that EA increased the total distance mice traveled in the open field (Figures 6 C,D). The pole climbing experiment showed that EA reduced the climbing experiment duration in mice (Figure 6E). The rod rotation test showed that EA increased the duration of mice on the rod rotation fatigue meter (Figure 6F). These results demonstrated that EA improves MPTP-induced body weight loss and locomotor activity decreasing.

### 3.7 EA Reduces MPTP-Induced Loss of Dopaminergic Neurons and the Activation of Microglia in a Mouse Model of PD

**FIGURE 7:**
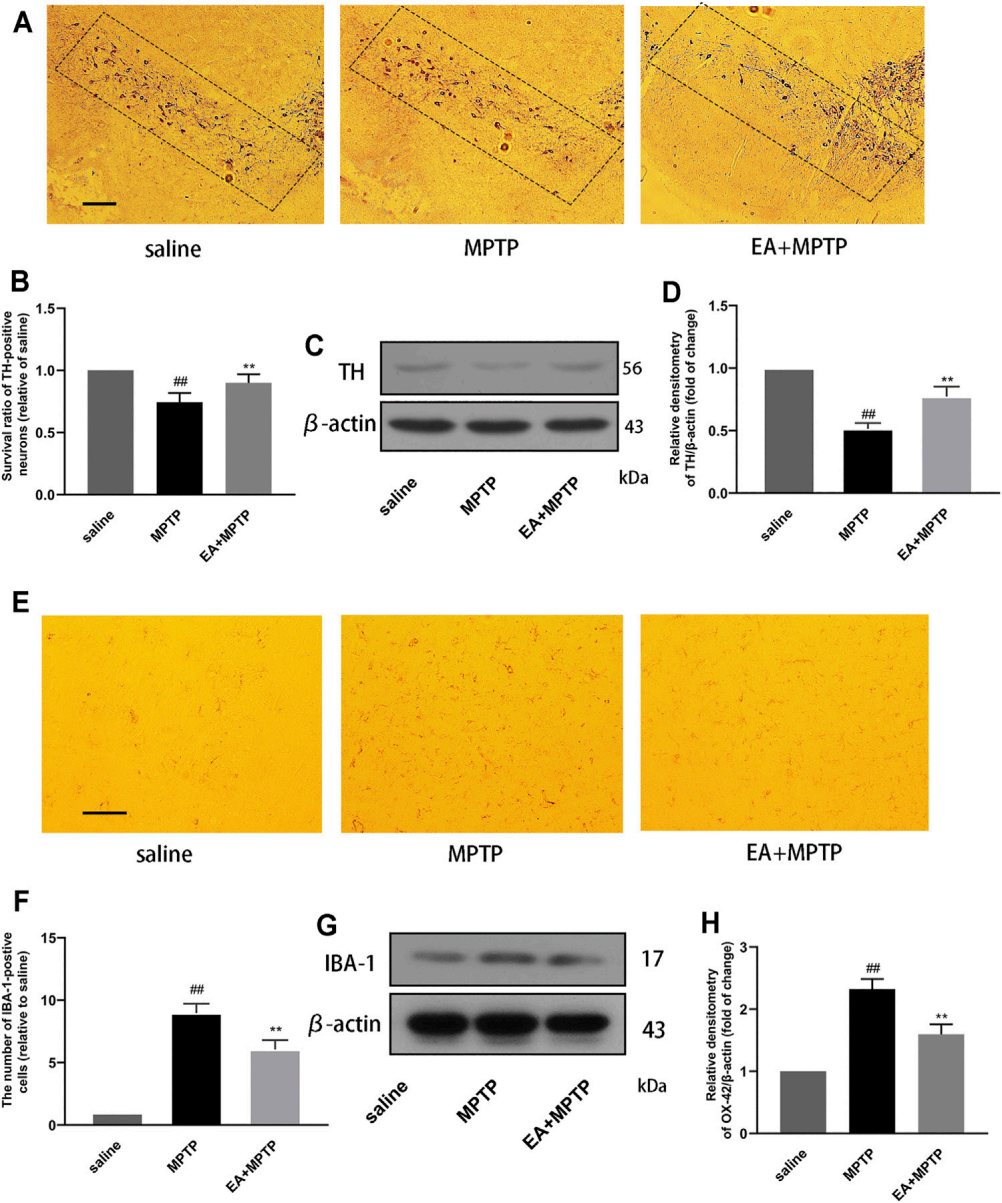
EA reduces MPTP-induced loss of dopaminergic neurons and the activation of microglia in a mouse model of PD. We obtained mouse midbrain tissue, formaldehyde fixation, and paraffin section. The number of TH-positive cells was detected by immunohistochemical assay (100 μm) **(A,B)**. The expression of TH protein in the midbrain region of mice was detected by Western blot assay **(C,D)**. The number of IBA-1–positive cells was detected by immunohistochemical assay (30 μm) **(E,F)**. The expression of IBA-1 protein in the midbrain region of mice was detected by Western blot assay **(G,H)**. Results are represented as means ± SD (*n* = 4). ^*##*^ *p <* 0.01 vs. saline group. **p < 0.01 vs. MPTP group.

The main pathological feature of PD is the damage of dopaminergic neurons in the substantia nigra of the midbrain. To clarify the role of EA, we examined the effect of EA (5 mg/kg) on the damage of dopaminergic neurons in the substantia nigra of the midbrain in mice. The results showed that EA improved MPTP-induced loss of dopaminergic neurons (Figures 7A,B). TH is a rate-limiting enzyme synthesized by dopaminergic neurons. We also detected the protein expression of TH in the midbrain by Western blot (Figures 7 C,D). The results demonstrated that EA inhibited the reduction of TH protein induced by MPTP.

The pathogenesis of PD is accompanied by excessive activation of microglia. In order to study the effect of EA, we tested the effect of EA on the activation of microglia. Immunohistochemical results showed that EA inhibited the number of IBA-1–positive cells in the substantia nigra (Figures 7E,F). Western blot results showed that EA inhibited the protein expression of IBA-1 in the mouse midbrain (Figures 7G,H). The results demonstrated that EA inhibited the activation of microglia.

### 3.8 EA Inhibits Inflammation in the MPTP-Induced Mouse PD Model

**FIGURE 8:**
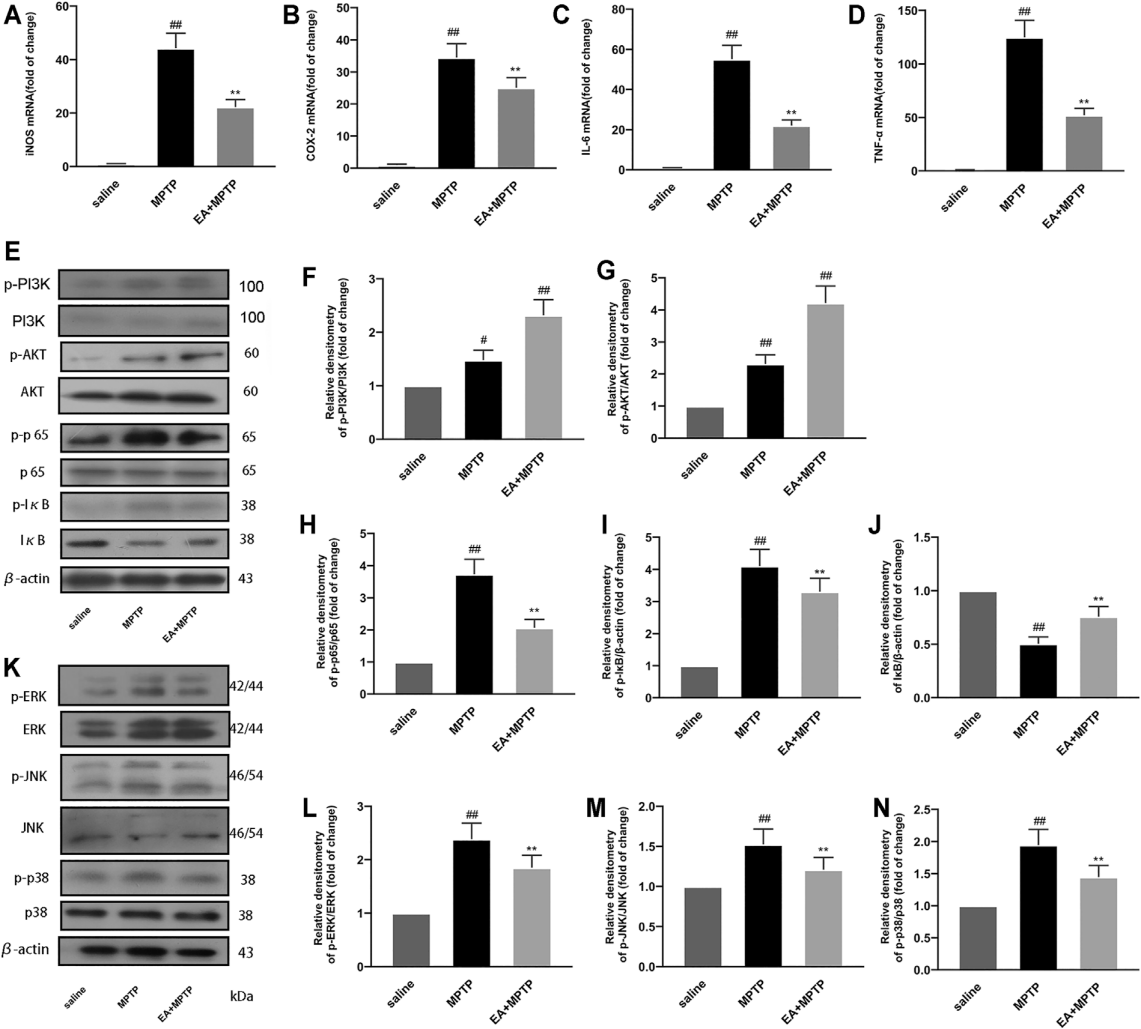
EA inhibits MPTP-induced inflammation in a mouse model of PD. We obtained mouse midbrain tissue. The mRNA expression of inflammatory mediators [iNOS **(A)**, COX-2 **(B)**, IL-6 **(C)**, and TNF-α **(D)**] was detected using quantitative PCR experiment. The protein expression of PI3K/Akt **(E–G)**, NF-κB **(E–J)**, and MAPK **(K–N)** pathways was detected by Western blot experiment. Results are represented as means ± SD (*n* = 4). ^*##*^ *p <* 0.01 vs. saline group. **p < 0.01 vs. MPTP group.

To further investigate the mechanism by which EA exerts its neuroprotective effect, we investigated the effects of EA (5 mg/kg) on mediators and inflammatory pathways in a PD mouse model. The results of fluorescence quantitative PCR showed that EA inhibited the mRNA expression of pro-inflammatory mediators (iNOS (Figure 8A), COX-2 (Figure 8B), IL-6 (Figure 8C), and TNF-α (Figure 8D). The results of Western blot showed that EA activated PI3K/Akt (Figures 8E–G) and inhibited NF-κB (Figures 8E,H–J) and MAPK (Figures 8K–N) signal pathways. These results demonstrated that EA inhibits MPTP-induced inflammation in the MPTP-induced mouse PD model.

## Discussion

PD, the second most prevalent neurodegenerative disorder affecting human health, is characterized by dopaminergic neuron damage in the substantia nigra of the midbrain, resulting in reduced dopamine content in the striatum, which leads to movement disorders in patients (Dauer and Przedborski, 2003; Hawkes et al., 2010). Therefore, the treatment of PD mainly focuses on increasing the number of dopaminergic neurons in the midbrain substantia nigra of the patient or enhancing the capacity of the remaining dopaminergic neurons to produce dopamine. In our experiment, we intraperitoneally injected MPTP into mice to establish a PD mouse model, and then studied the effects of EA on the motor behavior and dopaminergic neurons in the substantia nigra of PD mice. The results revealed that EA could inhibit MPTP-induced damage of dopaminergic neurons in the mice and improve their movement disorders. However, the mechanism by which EA protects neurons is unclear.

Studies have revealed that neuroinflammation is involved in the process of PD. When PD occurs, neurons are damaged, and the signal from the damaged neurons stimulates the body’s innate immune response, followed by the proliferation of immune cells that gather at the injured site to remove the damaged tissues and harmful factors. However, excessive immune cells and continuous inflammatory response lead to pro-inflammatory mediator accumulation, which further aggravates neuronal damage (Ishiki et al., 2017; Han et al., 2019; Qi et al., 2019). Therefore, inhibition of neuroinflammatory responses is of great significance in alleviating PD. In our experiment, we investigated the effect of EA on neuroinflammatory responses in MPTP-induced PD mice. The results revealed that EA inhibited the inflammatory response in the midbrain region of PD mice.

Microglia cells are the main effector cells of neuroinflammation (Ekdahl et al., 2009; Cameron and Landreth, 2010). BV2 is a microglia cell line cultured artificially (Henn et al., 2009). LPS is a Gram-negative bacterial cell wall component and can induce inflammatory responses (Huang et al., 1995; Peng et al., 2018). In our experiment, we stimulated BV2 cells with LPS to induce inflammatory response and surveyed the effect of EA on neuroinflammation. The results showed that EA inhibited the production of inflammatory mediators in BV2 cells. We used the supernatant of BV2 cells treated with EA and LPS to incubate two kinds of neuronal cell lines (SN4741 and SHSY5Y cells) and discovered that EA treatment improved the neurotoxicity mediated by microglia activation and played a neuroprotective role.

PI3K (phosphatidylinositol kinase) is a protein dimer composed of the regulatory subunit P85 and the catalytic subunit P110. When PI3K binds to growth factor receptors (such as EGFR), it changes the protein structure of Akt and activates it. PI3K activates or inhibits the activity of a series of downstream substrates through phosphorylation, thereby regulating cell proliferation, differentiation, apoptosis, and migration phenotypes (Gang et al., 2010; Alzahrani, 2019; Ghoneum and Said, 2019). In our experiment, we investigated the effect of EA on the PI3K/Akt pathway and found that EA activated the PI3K/Akt pathway. However, after inhibitors blocked the PI3K/Akt pathway, the inhibitory effect of EA on pro-inflammatory mediators was weakened to a certain extent. These results proved that EA inhibited the production of pro-inflammatory mediators by activating the PI3K/Akt signal pathway.

Nuclear factor-kappa B (NF-κB) and mitogen-activated protein kinase (MAPK) are two critical inflammatory pathways that regulate the transcription of inflammatory mediators after activation. NF-κB is composed of two subunits, p65 and IκB, in the cytoplasm. After activation, the IκB protein is separated and degraded by the two subunits, while p65 is phosphorylated into the nucleus and regulates the transcription of cytokines (Trickler et al., 2005; Mc Guire et al., 2013; Scott and Roifman, 2019). In our experiment, we investigated the effect of EA on the NF-κB pathway. Results showed that EA inhibited the phosphorylation of IκB and p65 and degradation of IκB. Three subunits are present downstream of the MAPK pathway: ERK, P38, and JNK (Wagner and Nebreda, 2009; Costa et al., 2016; Seger and Wexler, 2016). In our experiment, we surveyed the effect of EA on the MAPK pathway. Results showed that EA inhibited the phosphorylation of ERK, P38, and JNK. *In vivo* studies have also confirmed that EA activates the PI3K/Akt pathway and inhibits the NF-κB and MAPK signal pathways.

**FIGURE 9:**
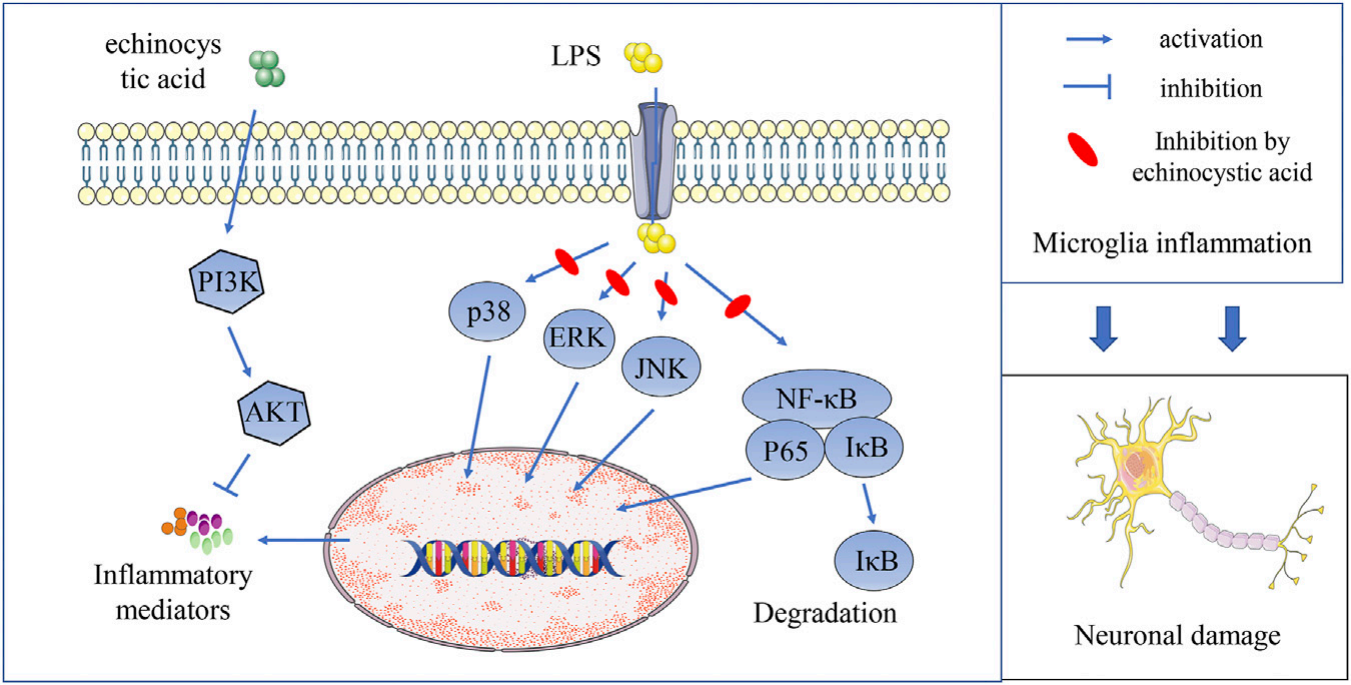
EA inhibits neuroinflammation and exerts neuroprotective effects by activating PI3K/Akt signaling and inhibiting NF-κB and MAPK signal pathways.

In conclusion, our study discovered that EA could improve PD symptoms in mice by activating PI3K/Akt and inhibiting NF-κB and MAPK signal pathways, inhibiting neuroinflammation and playing a neuroprotective role (Figure 9). EA as a plant extract with low side effects is economical and readily available, so it is expected to be further developed and utilized. Our study would contribute to further search for effective treatments for PD.

## Data Availability

The original contributions presented in the study are included in the article/Supplementary Material; further inquiries can be directed to the corresponding authors.
